# The Lure of Counterfactual Curiosity: People Incur a Cost to Experience Regret

**DOI:** 10.1177/0956797620963615

**Published:** 2021-01-13

**Authors:** Lily FitzGibbon, Asuka Komiya, Kou Murayama

**Affiliations:** 1 School of Psychology and Clinical Language Sciences, University of Reading; 2 Graduate School of Humanities and Social Sciences, Hiroshima University; 3 Research Institute, Kochi University of Technology

**Keywords:** information seeking, risk, decision making, emotions, rewards, open data, open materials, preregistered

## Abstract

After you make a decision, it is sometimes possible to seek information about how things would be if you had acted otherwise. We investigated the lure of this counterfactual information, namely, *counterfactual curiosity*. In a set of five experiments (total *N* = 150 adults), we used an adapted Balloon Analogue Risk Task with varying costs of information. At a cost, people were willing to seek information about how much they could have won, even though it had little utility and a negative emotional impact (i.e., it led to regret). We explored the downstream effects of seeking information on emotion, behavior adjustment, and ongoing performance, showing that it has little or even negative performance benefit. We also replicated the findings with a large-sample (*N* = 361 adults) preregistered experiment that excluded possible alternative explanations. This suggests that information about counterfactual alternatives has a strong motivational lure—people simply cannot help seeking it.

After one makes a decision, it is common to reflect not only on the outcome that was achieved but also on what might have been. For example, one might consider whether going to a party would have been more fun than staying home to work on a manuscript. These counterfactual comparisons can have negative emotional consequences; they can lead to the experience of regret (Loomes & Sugden, 1982). In the current study, we examined a commonly observed yet understudied aspect of counterfactual comparisons: the motivational lure of counterfactual information—*counterfactual curiosity* (FitzGibbon, Moll, Carboni, Lee, & Dehghani, 2019). Specifically, we found that people are so strongly seduced to know counterfactual information that they are willing to incur costs for information about how much they could have won, even if the information is likely to trigger negative emotions (regret) and is noninstrumental to obtaining rewards.

People frequently contemplate counterfactual alternatives to reality by mentally simulating possible alternative outcomes to past events. However, there are also occasions when these counterfactual considerations can be verified by seeking out the true outcome of previously rejected choice alternatives. For example, when shopping, after choosing a line at the checkout, we may find it irresistible to monitor the progress of another shopper in the adjacent line. In this way, we seek information about what would have happened had we made a different choice. Information seeking about counterfactual alternatives has been studied in human adults (Shani, Tykocinski, & Zeelenberg, 2008; Shani & Zeelenberg, 2007; Summerville, 2011; van Dijk & Zeelenberg, 2007), children (FitzGibbon et al., 2019), and rhesus macaques (Wang & Hayden, 2019).

Although counterfactual information can sometimes have instrumental value (Boorman, Behrens, & Rushworth, 2011), there are also examples of people simply wanting the counterfactual information for its own sake. Noninstrumental information seeking may be thought of as an expression of curiosity or a more general desire to resolve uncertainty (Gottlieb & Oudeyer, 2018). Counterfactual curiosity can be observed when the sought information cannot lead to better decision-making in the future, such as when outcomes are randomly generated in a gambling task. In the information-seeking literature, noninstrumental seeking behavior has been empirically demonstrated in a variety of ways. For example, people will incur small costs to receive information about upcoming rewards even though that information cannot be used to modify those rewards or improve decision-making (Bennett, Bode, Brydevall, Warren, & Murawski, 2016). Humans also sometimes feel a compulsion for potentially negative information. For example, people choose to expose themselves to various kinds of negative stimuli, including images depicting violence and physical harm (Oosterwijk, 2017) and the details of partners’ infidelity (Kruger & Evans, 2009). These kinds of behaviors are difficult to explain from traditional economic and decision-making theories and suggest that information itself may hold some strong motivational value, prompting organisms to approach the information without the reflection of its potential benefit and risk (FitzGibbon, Lau, & Murayama, 2020; Litman, 2005).

Despite the increasing number of empirical studies investigating the lure of curiosity, counterfactual curiosity has received little attention. In the literature of counterfactual comparisons, researchers have found effects of counterfactual comparisons in many areas of cognition, including reasoning (Smallman & Summerville, 2018), reward learning (Boorman et al., 2011), and moral judgment (Alicke, Buckingham, Zell, & Davis, 2008). However, in most of these studies, forgone information is either provided to or imagined by participants, so they cannot address the underlying motivation that prompts people to seek counterfactual information.

Only a limited number of studies have demonstrated that human adults will choose to seek noninstrumental information about forgone alternatives (Shani et al., 2008; Shani & Zeelenberg, 2007; Summerville, 2011; van Dijk & Zeelenberg, 2007). For example, Shani and Zeelenberg (2007, Experiment 4) asked participants whether they would check the numbers on a lottery ticket that they forgot to submit to see if they would have won the jackpot and found that participants reported that they would check. However, most of these studies relied on a scenario design, in which participants imagined having made the decision and then reported whether they would seek information about the alternative outcomes (Shani et al., 2008; Shani & Zeelenberg, 2007; Summerville, 2011, Experiments 2, 3, and 4). In addition, in all of the studies, participants were able to choose to see the forgone option without incurring costs. In such situations, information seeking about the alternative outcomes cannot inform us about the strong motivational value of forgone options and may even reflect participants’ random responding.

**Statement of Relevance**

Why is it that people want to know what might have been? This phenomenon of counterfactual curiosity is commonly observed, yet understudied. In this research, we conducted a set of experiments to test people’s motivation to find out how things could have turned out but did not. Using a simulated gambling situation, we found that people will actually incur costs to gain information about what they could have won, even though that information could not change the outcomes of the current or future gambles. They seek this information in spite of the possibility that it will make them feel bad about their choices. This research shows that information about what might have been has motivational salience—people are motivated simply to have the information. Motivational salience might also help to explain the fear-of-missing-out (FOMO) phenomenon. It seems that we just cannot resist learning about missed opportunities, social or otherwise.

In the set of experiments presented in this article, we addressed the motivational lure of counterfactual curiosity by exploring people’s willingness to seek information about what they could have won in a modified Balloon Analogue Risk Task (BART; Lejuez et al., 2002) when the information came free of cost (Experiment 1) as well as when participants had to incur a cost, such as money (Experiment 2), physical effort (Experiments 3 and 4), and time (Experiment 5). On each trial (see Fig. 1), participants pumped up a balloon to earn points and tried to avoid bursting the balloon by going beyond its randomly assigned safe limit. After seeing the trial’s outcome (“bank” or “bust”), participants were given the opportunity to seek information about the balloon’s limit to learn how much they could have won on that trial (see Fig. 1).

**Fig. 1.**
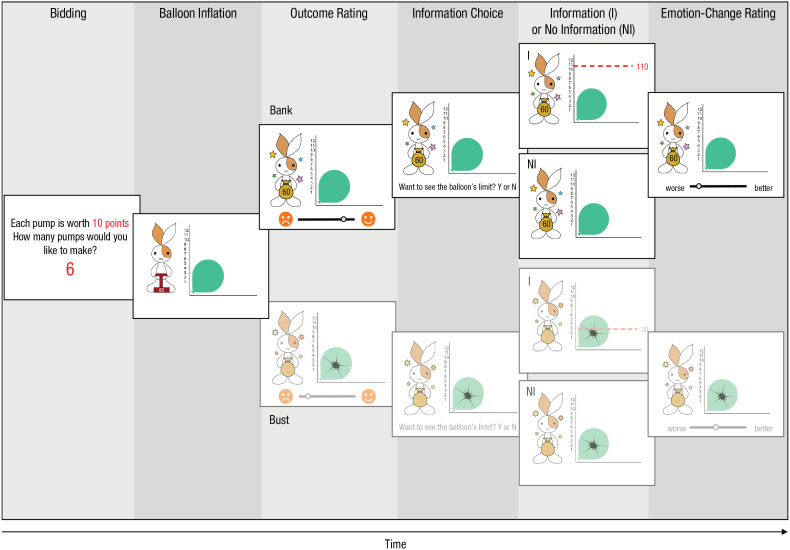
Experiment 1 trial sequence for bank and bust trials with and without information seeking. On bank trials, the balloon did not burst, and participants received points; on bust trials, the balloon broke, and participants received no points. Participants could then decide whether to see the actual limit the balloon could have been inflated on that trial. When this information was sought, a red dashed line indicated the safe limit of the balloon and the number of points that participants could have obtained. When this information was not sought, this red line was not shown. Participants made two ratings: first, how they felt after winning or losing points and, second, after seeking or not seeking information. Our main analyses focused on bank trials.

This task provides an ideal experimental setting to examine the motivational lure of counterfactual curiosity because (a) the balloon’s limit changes randomly across trials, meaning that postoutcome information about the balloon’s limit has little instrumental value in principle, and (b) after a bank outcome, participants were almost guaranteed to learn bad news (i.e., “I could have won more”) given that there was only a small chance that participants pumped the balloon just the right amount. This information is likely to lead to regret—a negative emotion focused on one’s own past action. Regret results from the comparison of an obtained outcome with a better outcome that would have been achieved if one had acted differently (Camille et al., 2004). By taking advantage of such features of the experimental task, we examined whether participants were willing to incur a cost to seek information about how much they could have won if they had pumped the balloon further. This information bore little instrumental utility and was likely to have a negative impact on their emotions (i.e., regret). We also explored further implications of the lure of counterfactual curiosity by investigating the downstream effects of seeking information about missed opportunities—whether people used the counterfactual information to adjust their behavior even if the information was almost noninstrumental. Because regret also has a strong motivational function to bias people’s decision-making behavior (Epstude & Roese, 2008), it is possible that information-seeking behavior in the current task ultimately led to increased risk taking (Büchel, Brassen, Yacubian, Kalisch, & Sommer, 2011). Thus, counterfactual curiosity may make people expose themselves to information that not only makes them feel bad but also will lead them to take further risks. We examined this possibility in an additional exploratory analysis.

## Main Study

### Method

#### Participants

One hundred fifty participants (100 female) were recruited for five experiments (30 participants for each). Table 1 contains sample characteristics and experimental manipulations for each experiment. Participants were recruited through either the Sona Systems (https://sona-systems.com) panel at the University of Reading or Prolific (https://prolific.co). Participants recruited through Sona Systems received course credit in return for their participation; participants recruited using Prolific were paid £2.50 for approximately 30 min of study participation plus a bonus of up to £1.50 dependent on task performance. The sample sizes were predetermined. On the basis of the work by Arend and Schäfer (2019), we determined that our total sample size (and the number of trials described below) would be sufficient (i.e., statistical power > 80%) to detect a small effect (i.e., β = 0.1) of trial-level predictors, which was the main focus of our analyses. Note that we were not primarily interested in the results of individual experiments or the effects of the different cost manipulations; we were interested in decision-making behavior that is not dependent on experimental context or the way we conceptualize cost. Thus, we conducted different experiments with slightly different procedures and integrated the results to make a stronger case for the generalizability of our findings (Westfall, Judd, & Kenny, 2015). For interested readers, however, results from individual experiments are available in Figures S5 to S8 in the Supplemental Material available online, and we also report an index (*I*^2^) that quantified the overall differences among the experiments (see the Data Analysis section). No participants were excluded from the analysis. All procedures were approved by the University of Reading’s School Research Ethics Committee (SREC; 2016-109-KM).

**Table 1.** Experimental Manipulations and Sample Characteristics for the Five Experiments in the Main Study

Experiment	Cost	Performance bonus	Recruitment site	Sample size	Mean age (years)	Age range (years)
Exp. 1	No cost	No	Sona	*N* = 30 (28 women)	19.90 (*SD* = 1.70)	18–26
Exp. 2	Money	Yes	Prolific	*N* = 30 (15 women)	34.62 (*SD* = 11.37)	20–68
Exp. 3	Physical effort	No	Sona	*N* = 30 (27 women)	19.61 (*SD* = 1.07)	18–22
Exp. 4	Physical effort	Yes	Prolific	*N* = 30 (15 women)	36.03 (*SD* = 13.50)	18–69
Exp. 5	Time (5 s)	Yes	Prolific	*N* = 30 (15 women)	38.27 (*SD* = 12.63)	18–62

Note: Sona refers to the undergraduate-student research-participation panel at the University of Reading. Prolific.co is an online participant-recruitment site.

#### Procedure

In all five experiments, participants completed two practice trials and 60 test trials of the BART with advance bidding. The five experiments had a very similar trial structure, and differences among experiments are described after the main procedure. An example trial sequence from Experiment 1 can be seen in Figure 1. Links to the experimental materials are available on OSF (https://osf.io/mtsqv/).

In the BART, participants inflated a balloon and earned points in proportion to the number of pumps they made. Importantly, each balloon had a randomly selected “safe limit”; if the balloon was pumped beyond this limit, then it would explode, and no points would be earned. On each trial, the participants were first shown the number of points that each pump was worth (pump value; this was randomly selected on each trial from a uniform distribution of 1–100). They then chose how many times to pump a balloon (number of pumps; between 1 and 12 pumps), which was followed by an animation showing a cartoon rabbit pressing a pump to inflate the balloon the number of times the participant chose (irrespective of the outcome). With each pump, the balloon was enlarged, and the number of points was displayed and updated. Then the outcome was revealed: The balloon either remained intact (outcome = bank) or burst (outcome = bust), signifying whether or not they had pumped the balloon beyond its safe limit. The safe limit was randomly selected on each trial from a uniform distribution between 0 (the balloon would burst after one pump) and 12 (the balloon would not burst after 12 pumps). Participants were explicitly informed in the instructions that the safe limit was determined randomly on every trial. When the outcome was revealed, the rabbit was depicted holding a bag that showed the points banked on the trial: the pump value multiplied by the number of pumps made on a bank trial and 0 points on a bust trial.

When the outcome was displayed, a visual analogue scale with pictures of a sad face on the left and a happy face on the right appeared below the rabbit and the balloon. Participants were asked to rate how they felt by dragging the marker along the scale. This rating, scored between −200 and 200, is referred to as the outcome rating. Participants were then given the opportunity to see the balloon’s safe limit (i.e., how far they could have pumped the balloon safely in that trial). If they chose to do so, the balloon’s safe limit was shown for 4,000 ms. This was depicted as a horizontal red line in the balloon area showing where the balloon could have been safely pumped to and the total point value associated with that limit (i.e., how much they could have won). If they chose not to view the balloon’s safe limit, participants still waited 4,000 ms before making the second rating (this was done to equate the time to the following emotion-change rating between when participants chose to see the limit and when they did not). After the 4,000 ms had elapsed, participants rated how their emotional state had changed on a visual analogue scale with the left anchor as “worse” and the right anchor as “better.” This rating, also scored between −200 and 200, is referred to as the emotion-change rating. The trial ended when this rating was submitted. The purpose of asking participants to rate how their emotions had changed was to allow the second rating to capture the full range of change even after maximally positive or negative emotion ratings after seeing the outcome (for a similar procedure, see O’Connor, McCormack, & Feeney, 2012). This measurement is aligned with a common definition of regret in the literature as a negative emotion experienced after learning that one could have had a better outcome if one had acted differently (Camille et al., 2004; Coricelli et al., 2005; Kahneman & Miller, 1986). We did not directly ask participants to rate their regret directly because this assessment may potentially bias ratings by eliciting demand characteristics.

To ensure the generalizability of the findings, we varied the cost of receiving information about the balloon’s safe limit and the conversion of earned points into a monetary bonus across the five experiments (see Table 1). In Experiment 1, there was no cost; in Experiment 2, participants paid 5 points to learn the balloon’s outcome; in Experiments 3 and 4, participants put in physical effort by making as many key presses as they could in a 4-s period; and in Experiment 5, participants incurred a 5-s time penalty after the trial. In Experiments 1, 2, and 5, if participants chose to view the balloon’s limit, then it was always shown to them. In contrast, in Experiments 3 and 4, there was a threshold of effort determined by an initial calibration phase and jittered randomly on each trial. If participants exceeded this threshold, then they were shown the balloon’s safe limit as described above. If they did not exceed the threshold (12% and 8% of trials in Experiments 3 and 4, respectively), then the words “try harder” were displayed for 4,000 ms before they made their second emotion rating. These try-harder trials were excluded from the analyses predicting the emotion-change rating because participants did not receive the information (try-harder trials were included in the analyses predicting information seeking reported in the Supplemental Material). In Experiments 2, 4, and 5, participants received a monetary bonus payment based on the points they earned in the task.

#### Data analysis

As noted earlier, we were interested in effects that are generalizable across different cost manipulations. Thus, we report the integrated results of the five experiments using a meta-analytic approach. We report all the experiments conducted to avoid publication bias, and our decision to conduct the five experiments was motivated to cover all of the potential cost manipulations (Ueno, Fastrich, & Murayama, 2016). We also report *I*^2^ to indicate the heterogeneity of the effects across experiments; *I*^2^ greater than 50% indicates substantial heterogeneity (Borenstein, Hedges, Higgins, & Rothstein, 2009). All analyses were conducted in the R programming environment (R Core Team, 2020). Further information about the packages used can be found in Table S1 in the Supplemental Material.

Our experimental design allowed us to analyze both bank and bust trials. The main analyses focused on bank trials. This is because bank trials gave us an optimal setting to test our primary research question—whether participants seek information that is likely to lead to negative emotional experiences (regret; see Büchel et al., 2011). On bank trials, information about the balloon’s limit primarily signals missed opportunities—how much more the participant could have won on each trial. As a result, information was expected to make participants feel worse because it was likely that they would learn that they could have won more rather than being “just right.” Bank trials are the primary focus of analyses in which similar tasks are used in the literature of regret (Brassen, Gamer, Peters, Gluth, & Büchel, 2012; Büchel et al., 2011). In contrast, on bust trials, it was expected that participants would experience regret only when they were very close to the balloon’s limit (a near miss; see Clark, Lawrence, Astley-Jones, & Gray, 2009). In the majority of the bust trials, on the other hand, they learned that they would not have won even if they pumped slightly fewer times; thus, we did not expect them to feel bad but, rather, to feel neutral or even a sense of relief that they probably would have gone bust anyway. Thus, our main analyses focused on bank trials. However, for the purpose of completeness, we followed the exact same analysis pipeline with bust trials, and the results are briefly reported later (full results are presented in the Supplemental Material).

For each experiment, we first examined the frequency of information-seeking behavior after bank trials. Then, to examine the emotional effects of information-seeking behavior, we built models predicting emotion-change ratings from information seeking using mixed-effects models with the *lme4* package (Bates, Mächler, Bolker, & Walker, 2015). Mixed-effects models allowed us to examine trial-level associations of the variable after taking into account the data dependency due to the nested structure of the data (trials were nested within participants). The model includes not only information-seeking behavior as the main independent variable but also other trial-level controlling variables (i.e., the point value of each pump [pump value] and the difference between the number of pumps made and the balloon’s limit [missed opportunity]) as well as their interactions with the information-seeking behavior. We first tested the model only with the main effects of information seeking and the controlling variables (Step 1) and then included their interactions with information seeking (Step 2). Emotion-change ratings were standardized (at the sample level; i.e., across participants and trials) before the analysis. Information seeking was effect coded (–1 = information not sought, 1 = information sought). The magnitude of the missed opportunity was kept in its original scale and mean centered within participants. The pump value was standardized at the sample level and then mean centered within participants. We specified all of the random slopes as well as random intercepts to appropriately control for Type I error rates. To aid model convergence, we reduced model complexity by forcing the correlation parameters of the random effects to zero (Matuschek, Kliegl, Vasishth, Baayen, & Bates, 2017). Some models nonetheless resulted in singular model fits (i.e., some of the random-effects variances were estimated as 0). We report the maximally specified models, described above, to maintain consistency across models. However, we also confirmed that the reported results are robust to the removal of random slopes terms to allow nonsingular fit from all the models.

Note that the number of pumps that participants made can be another controlling variable in our analyses, but this information is partly reflected in missed opportunities and thus suffers from the issue of multicollinearity. Therefore, this variable was not included in this analysis and the following path modeling (described below). However, to check the robustness of our findings, we repeated the analyses after replacing missed opportunities with number of pumps and confirmed that all of the main statistically significant results remained unchanged.

To determine the downstream effects of information seeking, we also conducted a multilevel path analysis. This model built on the previous analysis but added behavior adjustment and task performance on trial *t* + 1 as distal outcomes. More specifically, this model had emotion-change ratings on trial *t* and behavior adjustment on trial *t* + 1 as mediators between information seeking on trial *t* and performance on trial *t* + 1 (there were two outcomes: the outcome [bank or bust] and the number of points won). Like the previous analysis, this analysis included the pump value and magnitude of the missed opportunity on trial *t* as control variables along with their interactions with information seeking. Data from each experiment were analyzed with the *lavaan* package (Rosseel, 2012), with the nested structure of the data (trials nested within participants) being accounted for by calculating cluster-robust standard errors using *lavaan.survey* (Oberski, 2014). We also computed model fit, which indicates the appropriateness of the paths that we omitted from the model (e.g., a direct path from information seeking to behavior adjustment). Our model showed good fit to the data in most cases (average comparative fit index = 1.00, range = .98–1.00; average Tucker-Lewis index = 0.96, range = 0.89–1.02; and average root mean square error of approximation = .037, range = .000–.074), but we also report the parameter estimates from the saturated model (i.e., a model that estimated all the direct paths as well as mediation paths) in Tables S6 and S8 in the Supplemental Material. The unstandardized parameter estimates from these models (i.e., betas and standard errors) were then submitted to random-effects meta-analyses using the metafor package (Viechtbauer, 2010) to integrate individual model coefficients from the five experiments.

All data are available on OSF at https://osf.io/mtsqv/.

### Results

#### Frequency of information-seeking behavior

Descriptive statistics of the task performance in each of the five experiments can be found in Table 2. Participants were fairly conservative across the five experiments, pumping the balloon an average of 4.97 times out of 12. The optimal strategy for maximizing points would be to pump 6 or 7 times on every trial (Lejuez et al., 2002), but participants did not seem to be aware of that strategy. This conservative performance led to more bank than bust trials (*M* = 62%, *SE* = 6%). Importantly, overall, across the five experiments, participants sought information on nearly half of these bank trials (*M* = 46%, *SE* = 9%). The meta-analysis of the mean proportion of trials on which information was sought revealed significant heterogeneity between the experiments (*I*^2^ = 89%). Observing the proportion of information seeking across the five experiments reveals that information seeking was highest (*M* = 67%, *SE* = 7%) in Experiment 1 when it incurred no cost and was lowest (*M* = 18%, *SE* = 5%) in Experiment 2 when it incurred a monetary cost. Despite the heterogeneity, the probability of information seeking was significantly different from zero across all five experiments (all *p*s < .001). Investigation of the trial-level factors that predicted information seeking can be found in the Supplemental Material.

**Table 2.** Descriptive Statistics of Task Performance for the Five Experiments in the Main Study

Experiment	Mean number of pumps	Mean proportion of trials with bank outcome	Mean proportion of information seeking after bank outcome
Exp. 1	5.11 (0.23)	.60 (.02)	.67 (.07)
Exp. 2	5.09 (0.21)	.60 (.02)	.18 (.05)
Exp. 3	4.88 (0.18)	.64 (.02)	.56 (.06)
Exp. 4	4.86 (0.23)	.62 (.02)	.46 (.08)
Exp. 5	4.95 (0.24)	.62 (.02)	.44 (.08)
Integrated results	4.97 (0.09)	.62 (.01)	.46 (.09)
Heterogeneity (*I*^2^)	0%	3%	89%^a^

Note: Standard errors are given in parentheses.

a This value indicates significant heterogeneity among experiments at an alpha of .05.

#### Emotional experience

**Fig. 2.**
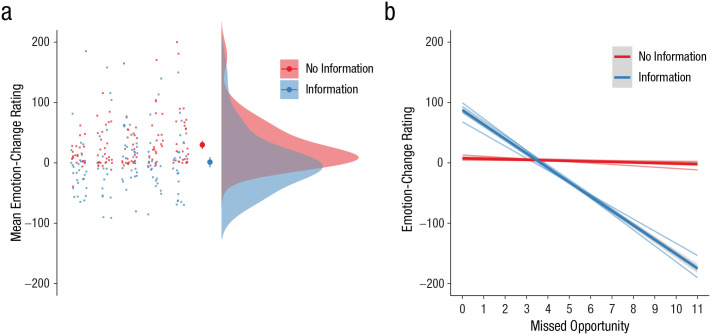
Effects of information on emotion-change ratings. In the raincloud plot (a), each column of small dots represents data from one experiment. Each small dot represents a participant’s mean emotion-change rating on trials in which they did (blue) and did not (red) seek information. The larger dots represent the means for the whole sample; error bars represent 95% confidence intervals. The shaded regions indicate the density of the data. The linear effects of missed opportunity on emotion-change ratings (b) are shown for trials on which participants did and did not seek information. Thicker lines represent the overall effects; thinner lines represent effects for each of the five experiments. The shaded area around the thicker line represents the standard error. Emotion-change ratings were mean centered within participants before plotting.

To examine whether seeking information after bank trials would generally lead participants to feel worse, we conducted mixed-effects modeling predicting participants’ emotion-change ratings from information seeking (–1 = information not sought, 1 = information sought), pump value, and missed opportunity (i.e., the difference between the number of pumps and the balloon’s limit; see Table 3, Step 1). Confirming our expectation, results showed that participants felt significantly worse after receiving information about the balloon’s limit than after rejecting that information (*b* = −0.163, *z* = −6.71, *p* < .001, 95% confidence interval, or CI = [−0.211, −0.115]; for average emotion-change ratings for each participant in each experiment after seeking and not seeking information, see Fig. 2a). This suggests that participants experienced regret when they learned how far they could have pumped the balloon. The effect had little heterogeneity across experiments (*I*^2^ = 0%), indicating the generalizability of the findings regardless of the cost manipulation. There was no significant main effect of the pump’s value, but there was a negative main effect of the missed opportunity (see Table 3, Step 1).

**Table 3.** Integrated Results in Models Predicting Emotion-Change Ratings From Information Seeking, Pump Value, and Missed Opportunity Across the Five Experiments in the Main Study

Model	Step 1: main effects					Step 2: main effects and interactions				
	*b*	*SE*	*z*	*p*	*I* ^2^	*b*	*SE*	*z*	*p*	*I* ^2^
Information seeking	−0.163	0.024	−6.71	< .001	0%	−0.173	0.025	−6.83	< .001	0%
Pump value	0.015	0.031	0.49	.625	51%	0.000	0.020	0.00	.998	0%
Missed opportunity	−0.142	0.023	−6.04	< .001	78%	−0.163	0.008	−21.04	< .001	14%
Information Seeking × Pump Value						−0.125	0.020	−6.08	< .001	0%
Information Seeking × Missed Opportunity						−0.144	0.012	−11.71	< .001	68%

Why did participants feel worse after seeing the information? To examine the potential mechanism, we added the interaction terms with information seeking to the model (see Table 3, Step 2). The main effect of information seeking remained significant (*b* = −0.173, *z* = −6.83, *p* < .001, 95% CI = [−0.222, −0.123], *I*^2^ = 0%). Importantly, the analysis showed that this main effect was qualified by a negative interaction with missed opportunity (see Fig. 2b; *b* = −0.144, *z* = −11.71, *p* < .001, 95% CI = [−0.168, −0.120], *I*^2^ = 68%). The magnitude of the effect showed heterogeneity across the experiments, but a significant negative interaction was observed across all experiments (all *p*s < .001; see Fig. 2b). Simple-slopes analyses suggest that when participants sought information, there was a large and significant effect of missed opportunity: the larger the missed opportunity, the worse they felt (*b* = −0.308, *z* = −16.35, *p* < .001, 95% CI = [−0.345, −0.271], *I*^2^ = 73%). These results indicate that the negative experience after information seeking was driven by the sense of missed opportunity—findings consistent with the literature on regret (e.g., Brassen et al., 2012). Participants felt positive when they were “just right” (see Fig. 2b), and there is a possibility that participants were motivated to seek information to have those positive experiences. However, the magnitude of positive emotion is small compared with that of the overall negative emotional experiences. In addition, exploratory analysis (see the Supplemental Material) showed that this positive emotion did not increase participants’ likelihood of seeking information on the next trial, suggesting that positive emotional experiences did not work as an incentive for information-seeking behavior in the next trial. In contrast to when participants sought information, when they did not seek information, there was a much smaller, yet still statistically significant, negative association between missed opportunity and emotion-change ratings (*b* = −0.012, *z* = −2.86, *p* = .004, 95% CI = [−0.021, −0.004], *I*^2^ = 18%). The smaller effect is not surprising because participants were unable to perceive missed opportunities when information was not sought. The significant relationship even when information was not sought may be due to the negative relationship between the number of pumps and the size of the missed opportunity—when participants made more pumps (and so won more), there was less room for a large missed opportunity.

There was also a significant negative interaction between pump value and information seeking (*b* = −0.125, *z* = −6.08, *p* < .001, 95% CI = [−0.165, −0.085], *I*^2^ = 0%). Simple-slopes analysis suggests that when participants did not seek information, the pump value positively predicted emotion-change ratings—because they won more points (*b* = 0.125, *z* = 5.17, *p* < .001, 95% CI = [0.077, 0.172], *I*^2^ = 0%). In contrast, when they did seek information, the value had a negative effect—they missed out on more points (*b* = −0.116, *z* = −4.37, *p* < .001, 95% CI = [−0.169, −0.064], *I*^2^ = 0%).

#### Downstream effects of information seeking

**Fig. 3.**
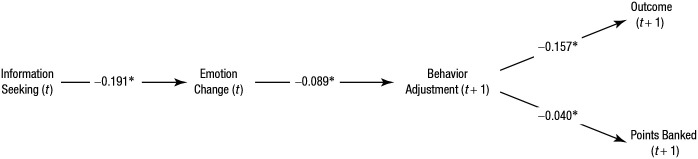
Path model demonstrating the effect of information seeking on emotion-change ratings (positive value means feeling better), behavior adjustment, and next-trial performance. Values shown are unstandardized coefficients. Asterisks indicate significant paths ( *p* < .05). The main effects of missed opportunity and pump value, along with their interactions with information seeking, were included as control variables.

To determine whether seeking information affected participants’ downstream behavior and performance, we conducted a multilevel path analysis (structural equation model; see Fig. 3). We aimed to once again show how information seeking affected participants’ emotional state and that this has consequences for subsequent behavioral change and performance. We modeled a path from information seeking via emotion change and behavioral adjustment on the next trial (positive value means becoming more risky after the current bank trial) to the outcome of the next trial (both the probability of a bank outcome and the number of points that participants banked). As in the previous analyses, we controlled for the missed opportunity and the pump value as well as the interaction between these and information seeking (these control variables are not shown in Fig. 3; for all the model parameters, see Table S5 in the Supplemental Material).

We once again showed that information seeking had a negative effect on emotion-change ratings overall (it made participants feel worse; *b* = −0.191, *z* = −6.13, *p* < .001, 95% CI = [−0.252, −0.130], *I*^2^ = 0%). Consistent with the previous results, this effect was qualified by the interactions with the missed opportunity (*b* = −0.656, *z* = −10.65, *p* < .001, 95% CI = [−0.777, −0.536], *I*^2^ = 0%) and pump value (*b* = −0.137, *z* = −5.33, *p* < .001, 95% CI = [−0.188, −0.087], *I*^2^ = 0%), but on average, information seeking made participants feel worse.

The next step of the path was between the emotion-change rating and the adjustment of the number of pumps made on the next trial. The worse participants felt, the riskier they became on the next trial (*b* = −0.089, *z* = −4.86, *p* < .001, 95% CI = [−0.125, −0.053], *I*^2^ = 0%). This behavioral adjustment had a negative effect on both the probability of a bank outcome (*b* = −0.157, *z* = −22.69, *p* < .001, 95% CI = [−0.170, −0.143], *I*^2^ = 0%) and the number of points banked (*b* = −0.040, *z* = −2.81, *p* = .005, 95% CI = [−0.069, −0.012], *I*^2^ = 0%) on the next trial. This suggests that participants’ adjustment in response to missed opportunities might have backfired on average. Note that all of these critical paths showed little heterogeneity across the experiments, suggesting the robustness and generalizability of the results over the different cost manipulations. We also tested the significance of the mediation effect from information seeking to the next trial outcome and the next trial points and found that mediation effects for the outcome were negative and statistically significant (*b* = −0.003, *z* = −3.42, *p* = .001, 95% CI = [−0.004, −0.001], *I*^2^ = 0%), although the effect size was very small. On the other hand, the mediation effect for the points banked was negative but did not reach statistical significance (*b* = −0.000, *z* = −1.43, *p* = .152, 95% CI = [−0.001, 0.000], *I*^2^ = 0%). These results suggest that information seeking had negative or little benefit on participants’ performance on the next trial.

#### Supplementary analysis and mathematical modeling on the noninstrumentality of the information

**Fig. 4.**
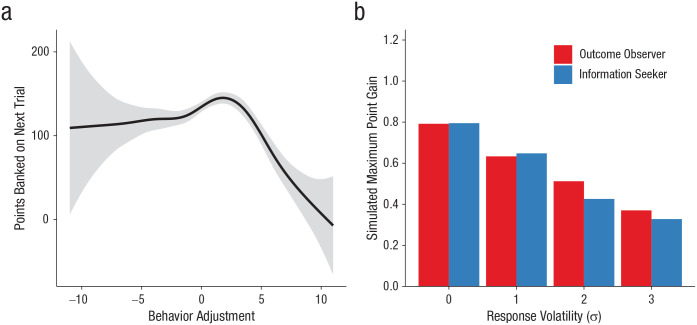
Detailed analysis and simulation of the potential instrumentality of information in the current task. The smoothed relationship between behavior adjustment and subsequent points banked on the next trial (a) is shown across all five experiments. The gray area around the line represents the standard error. The results of mathematical simulations comparing adjustments based on the outcome of the trial alone or the missed opportunity are shown in (b). The graph shows the maximum point gain of two types of participant as a function of the magnitude of response volatility. After the outcome is revealed, participants always either seek information about the balloon’s limit (information seekers) or decline to see the balloon’s limit (outcome observers).

Our data showed that participants were generally conservative in pumping the balloon (mean pumps = 4.97), and as noted earlier, performance on this task (in terms of points won) could be optimized by consistently selecting six or seven pumps across the trials (Lejuez et al., 2002). These facts imply that although we found an overall negative linear relationship between behavioral adjustment and obtained points, participants might have slightly benefited from being riskier after information-seeking trials. To precisely understand the behavioral consequence of information seeking in our task, we pooled the data from the five experiments and plotted the potential nonlinear relationship between behavioral adjustment and obtained points in the next trial using a generalized additive model (Hastie & Tibshirani, 2014; see Fig. 4a). As expected, when behavioral adjustment was small and positive (i.e., being slightly riskier), we observed a small performance gain. However, the performance dramatically decreased when positive performance adjustment was more than a few pumps, producing an overall negative relationship between performance adjustment and obtained points. The plot suggests that although an inherent feature of our task (i.e., there is an optimal strategy) brought some performance benefits after minor behavioral change, such a benefit is minimal and was offset by a large loss after bigger behavioral change.

In addition to the empirical investigation, mathematical simulations were also conducted to objectively evaluate whether the current task has an inherent structure that would benefit people who seek information. More specifically, we compared two types of hypothetical participants—those who always seek information (information seekers) and those who observe the outcome (bank or bust) but always decline to see the balloon’s limit (outcome observers). In this modeling, information seekers adjust their pumps in the next trial in proportion to the difference between the number of pumps they made and the balloon’s limit that they have seen. For example, when they learned that they could have pumped *k* more, they increased their pumps in the next trial by *k* × α_i_ + *e* pumps, where α_i_ is a parameter that represents the magnitude of adjustment for information seekers (0 < α_i_ < 1), and *e* is response volatility (noise), with *e* ~ *N*(0, σ^2^). On the other hand, outcome observers knew whether they won or lost only in the last trial, and they adjusted their pumps in the next trial in proportion to the difference between the number of pumps they made and the minimum or maximum possible limit of the balloon (i.e., 0 or 12) for bust and bank trials, respectively. For example, when they pumped 4 times and learned that they banked, they increased their pumps in the next trial by 8 × α_o_ + *e* pumps, where α_o_ is a parameter that represents the magnitude of adjustment for outcome observers (0 < α_o_ < 1). We randomly generated these parameter values in these models and compared their performance with that of a hypothetical baseline model in which participants do not make use of any feedback information to determine the number of pumps (i.e., Gaussian random-walk model). We simulated 10,000 of each type of hypothetical participant (with 60 trials each) with four different levels of response volatility, σ^2^ = {0^2^, 1^2^, 2^2^, 3^2^}. We then computed the maximum point gain for each type of hypothetical participant, which represents the maximum point benefit that they can gain by taking these strategies (i.e., information seeking or outcome observation) across the parameter space of α_i_ and α_o_ in comparison with the baseline model.

Figure 4b shows the maximum point gain of these two types of participant as a function of the magnitude of response volatility (for the optimal α_i_ or α_o_ value that maximizes task performance, see Table S4 in the Supplemental Material). Note that the maximum point gain is the average point benefit per trial, and for one trial, participants could receive between 0 and 12 points. As can be seen from the figure, information seekers benefit little more than outcome observers, indicating that adjusting pumps on the basis of information about the balloon’s limit provides little utility to maximize their performance in the current task, in comparison with just adjusting their pumps on the basis of the outcome (i.e., bank or bust) information (the benefit of information seeking over observing the outcome on maximum point gain was between 0.014 and −0.086). These results demonstrate the noninstrumentality of the information in the current task.

#### Analyses with bust trials

As noted earlier, with the current task structure, bank trials provide us with the best situation to test the seductive lure of counterfactual information because participants are more likely to experience regret after bank than bust trials. However, for the purpose of completeness, we repeated the same set of analyses with bust trials (full reports are available in the Supplemental Material). On average, participants sought information slightly less frequently in bust trials than bank trials (*M* = 43%, *SE* = 8%). As we expected, the effect of information seeking on emotion change was weaker or even nonsignificant (e.g., *b* = −0.066, *z* = −1.31, *p* = .190, 95% CI = [−0.164, 0.033]) when all the covariates and their interactions were included in mixed-effects modeling. On the other hand, all other effects were well replicated, including those in the path model. Similar to the pattern observed in bank trials, the relationship between the missed-opportunity and emotion-change ratings after information seeking was negative (*b* = −0.120, *z* = −2.29, *p* = .022, 95% CI = [−0.223, −0.017], *I*^2^ = 91%; see Fig. S1b in the Supplemental Material). Participants felt bad when there was a near miss, suggesting that they may also have experienced regret when they learned that they could have banked if they had pumped a little less. In contrast, they felt better when the limit was further from the number of pumps they made, suggesting that they experienced relief because they would not have won, even if they had pumped a little less. These results suggest that emotional responses to information after bank and bust trials may be supported by similar psychological processes, except that bust trials do not strongly elicit regret in comparison with bank trials.

## Replication Study

One limitation of this study is that although the information carried little instrumental utility (as shown in our analyses), participants may have erroneously believed that information about the balloon’s limit could improve their future performance. For example, they may have sought to gain a better understanding of the distribution of the balloon’s limit. In other words, participants may have sought information not because they were curious but because they believed that it bore some instrumental utility. To address this issue, we developed a modified version of the task in which participants completed only one critical trial after which they could seek information about how much they could have won. Because there were no further trials following the critical trial, it was extremely clear to participants that information seeking in the critical trial could in no way help improve their future task performance. In this way, we greatly reduced the possibility that participants would perceive utility from the information. If information seeking in the previous five experiments was largely driven by the expected utility of the information, then we would at least expect to see reduced information seeking. In contrast, if information seeking was not primarily driven by expected utility, then we would expect that people would still suffer a cost to seek it despite the clear nonutility of the information and its likely deleterious effect on mood.

### Method

#### Participants

A total of 361 participants took part in this study (199 women; mean age = 34.29 years, *SD* = 11.97, range = 18–77). Only participants who banked on the critical trial (*n* = 216, 59.83%) were given the opportunity to seek information about the balloon’s limit. Participants were recruited from Prolific (https://prolific.co) and completed the study online. Participants were paid £1 for approximately 10 min of participation. In addition, participants could earn up to £2.40 bonus on the critical trial. The average bonus (for those who banked) was £1.01. Those who went bust did not receive a bonus payment.

The sample size was predetermined. On the basis of the results of Study 1, we expected to see a medium to large effect of counterfactual information seeking on emotion ratings. On the basis of pilot data from 18 participants, we expected an uneven distribution between those who sought and those who did not seek information. We used G*Power (Version 3.1; Faul, Erdfelder, Lang, & Buchner, 2007) to determine the sample size required for the between-subjects, two-tailed *t* test with uneven groups. We used the following parameters: Cohen’s *d* = 0.5; α = .05; power = 80%; allocation ratio N2/N1 = 0.25. This gave a minimum sample of 200 participants. In a pilot study, eight out of 18 participants went bust; we thus increased our sample by an additional 160 participants to achieve the required number of participants who did not go bust. The design and hypotheses of Study 2 were preregistered (https://osf.io/yab8q/).

#### Procedure

The task was based on the one used in Study 1. As before, the balloon’s limit on each trial was sampled from a uniform distribution between 0 and 12. The value did not vary but was fixed at 20 pence per pump. In the new procedure, participants were given instructions explaining the task and completed a practice round “to get a feel for how the game works,” which consisted of 10 BART trials (as described in the main study). They rated their emotion after learning each outcome on a visual analogue scale with sad and happy face anchors (as used in the main study). In these 10 practice trials, participants were not given the option to see the balloon’s limit. After the 10 practice trials, participants received the instructions for the main experiment—that there is one last critical round in which they could earn a monetary reward (20 pence per pump). They then completed one critical round of the BART. Because our primary interest was in bank trials, we decided to offer information only to those participants who banked the money on the critical trial. If participants went bust on this final round, then they did not get a bonus payment, and they were not offered the opportunity to see the balloon’s limit. If they banked the money, then after rating their emotions about the outcome, they were asked whether they wanted to see the balloon’s limit of the critical trial. We used waiting time as a cost, as in Experiment 5 of the main study; participants were able to see the limit of the balloon if they were willing to wait 30 s before receiving a completion code to receive the compensation for the study. Willingness to incur this cost in return for information about the balloon’s limit would suggest that participants were not simply seeking the information to liven up the task, because it would substantially extend the length of the task with a long and boring wait for the completion code.

If they chose to see the information, then they saw an animation of the balloon inflating to its randomly determined limit and were presented with information about how much money they could have won on the trial. If they chose not to receive the information, then they continued to observe their obtained outcome for the same amount of time as if they had chosen to view the information so that the delay between the ratings was the same whether or not participants sought the information. Participants then rated their emotions a second time using the visual analogue scale with sad and happy face anchors. Note that, unlike in the main study, we assessed the current emotion rather than directly asking about participants’ “change of emotions” and used the change score from the first rating to the second rating as the index of regret. This departure from the method employed in Study 1 was made to address the possibility that asking people to report how their emotions had changed would encourage them to overestimate change compared with simply reporting their emotions twice.

### Results

Of the 216 participants who banked on the critical last trial, 153 (71%, 95% CI = [64%, 77%]) opted to take a 30-s time cost to see how much they could have won. Participants actually sought information more frequently in this one-shot experiment than in the multitrial experiment that also used a time cost (Experiment 5; 44%, 95% CI = [29%, 60%]). Although an exact statistical test for the comparison of the proportions between Experiment 5 and the replication experiment is difficult because of the different trial designs (i.e., in the replication study, each participant has only one binary value), 95% CIs for the rate of information seeking did not overlap, suggesting a reliable difference.

Participants who sought information reported significantly greater reductions in mood between the emotion ratings (*M* = −113.03, *SE* = 10.45) than those who did not seek information (*M* = −20.68, *SE* = 8.29), *t*(204.74) = −6.93, *p* < .001, *d* = −0.81, 95% CI = [−1.11, −0.50] (Welch’s *t* test; for a graphical representation of participants’ emotion ratings, see Fig. S4 in the Supplemental Material). These results demonstrated that our main findings were robust even if it was very clear to participants that the information carried no instrumental utility.

To better understand the role that information played in participants’ mood, we developed a linear model predicting participants’ standardized emotion-rating change from the interaction between information seeking (effect coded; −1 = information not sought, 1 = information sought) and the missed opportunity (mean centered) as well as their main effects. This exploratory analysis was not preregistered. Again, seeking information had a significant negative effect on the second emotion rating, *b* = −0.378, *t*(212) = −6.28, *p* < .001, 95% CI = [−0.497, −0.259]. There was also a negative effect of missed opportunity, *b* = −0.115, *t*(212) = −4.74, *p* < .001, 95% CI = [−0.163, −0.067]. However, there was a significant interaction between the size of the missed opportunity and information seeking, *b* = −0.121, *t*(212) = −5.01, *p* < .001, 95% CI = [−0.169, −0.074]. Simple-slopes analyses revealed that there was no effect of the missed opportunity when information was not sought, *b* = 0.006, *t*(212) = 0.16, *p* = .873, 95% CI = [−0.074, 0.087]. In contrast, after participants sought information, the larger the missed opportunity observed, the worse participants felt, *b* = −0.236, *t*(212) = −8.99, *p* < .001, 95% CI = [−0.288, −0.184]. These results again replicated our findings in the main experiments and demonstrated that our findings were robust to changes in how participants’ emotion was measured.

## General Discussion

In a set of five experiments using different costs of seeking information, participants frequently sought information about what they could have won in a sequential risk-taking task, despite that information having a negative impact on their mood. Reported regret caused by information seeking further led participants to display more risky behavior in the next trial, despite such behavioral adjustment having little or even negative performance implications. The results were replicated in a large-sample preregistered study even when the potential for participants to perceive utility of the information was greatly reduced, rendering it unlikely that information seeking in earlier experiments was driven purely by expected utility. These findings demonstrate that counterfactual curiosity, the desire for information about what might have been, has a strong motivational lure.

Instances of seeking negative information are puzzling because they contradict our notion that humans are rational decision makers who seek out positive emotional experiences and avoid negative ones. It seems counterintuitive that people would want to seek information that inevitably makes them feel bad. Indeed, there is a growing body of literature documenting the avoidance of information that might bring bad news or disconfirm cherished beliefs (Charpentier, Bromberg-Martin, & Sharot, 2018; Hertwig & Engel, 2016; Sweeny, Melnyk, Miller, & Shepperd, 2010). However, as the current studies demonstrate, there are also situations (such as after gambling episodes) in which people put themselves at risk of mood reduction simply to resolve uncertainty. This kind of information-seeking behavior is also part of the fear-of-missing-out (FOMO) phenomenon. For example, after deciding which of two parties to attend one evening, you might be tempted to seek information about the other party by looking at social media, even though you are having a good time and there is some chance that you will discover that the other party was even more fun (Przybylski, Murayama, DeHaan, & Gladwell, 2013).

One explanation for seeking negative information is that people may also find it interesting to test their emotional responses—a mechanism that might also underlie so-called morbid curiosity (Oosterwijk, 2017). Counterfactual information of the kind sought in the current experiments may be desirable because it has high personal relevance—it relates to decisions that one has made in the recent past (Golman & Loewenstein, 2018; Murayama, FitzGibbon, & Sakaki, 2019). People’s desire for information about their own performance is known to be strong enough to overcome cognitive biases such as inequality aversion (Alós-Ferrer, García-Segarra, & Ritschel, 2018). Thus, opportunities to learn about oneself and the actual and counterfactual consequences of one’s decisions may have powerful motivational status.

Future research can test the importance of personal relevance by manipulating the feeling of agency that the participant has over decisions. Manipulations of agency have powerful effects on people’s emotional evaluation of events (Coricelli et al., 2005), so it is intuitive that a sense of agency would also increase curiosity about outcomes and forgone alternatives of the past events. Determining how personality traits such as intolerance of uncertainty and sensation seeking relate to counterfactual curiosity is another important step for understanding individual differences in information seeking of this kind.

One limitation of the current study is that it is unknown whether the observed instances of not seeking information were driven by active avoidance of or passive indifference toward the information. Charpentier et al. (2018) devised a method to distinguish these motivations by presenting information about investment outcomes with .5 probability free of charge. Participants could then pay to shift the probability of either receiving or not receiving information, and in doing so, the researchers were able to dissociate active avoidance and passive ignorance. Future studies employing a similar design would elucidate whether choices not to seek counterfactual information are instances of avoidance or indifference and whether participants differ in the extent to which they seek, are indifferent to, or avoid information.

## Supplemental Material

sj-docx-1-pss-10.1177_0956797620963615 – Supplemental material for The Lure of Counterfactual Curiosity: People Incur a Cost to Experience Regret Click here for additional data file. Supplemental material, sj-docx-1-pss-10.1177_0956797620963615 for The Lure of Counterfactual Curiosity: People Incur a Cost to Experience Regret by Lily FitzGibbon, Asuka Komiya and Kou Murayama in Psychological Science

## Transparency

*Action Editor:* Marc J. Buehner

*Editor:* D. Stephen Lindsay

*Author Contributions*

L. FitzGibbon and K. Murayama developed the study concept. All the authors contributed to the study design. Testing was conducted by L. FitzGibbon, and L. FitzGibbon analyzed and interpreted the data under the supervision of K. Murayama. L. FitzGibbon drafted the manuscript, and K. Murayama and A. Komiya provided critical revisions. All the authors approved the final manuscript for submission.

**Open Practices:** All data, analysis code, and materials for the main study and the replication study have been made publicly available via OSF and can be accessed at https://osf.io/mtsqv/. The design and analysis plan for the replication study were preregistered at https://osf.io/yab8q; an additional analysis that was not preregistered is discussed in the text. The five experiments in the main study were not preregistered. This article has received the badges for Open Data, Open Materials, and Preregistration. More information about the Open Practices badges can be found at http://www.psychologicalscience.org/publications/badges.
