# Supplementary material for: The Lure of Counterfactual Curiosity: People Incur a Cost to Experience Regret
Source: Psychol Sci. 2021 Jan 13;32(2):241–55. doi: 10.1177/0956797620963615 (PMC7883003; doi:10.1177/0956797620963615)
Supplement: sj-docx-1-pss-10.1177_0956797620963615 – Supplemental material for The Lure of Counterfactual Curiosity: People Incur a Cost to Experience Regret [file sj-docx-1-pss-10.1177_0956797620963615.docx]

# Supplementary Online Materials

## R package versions and references

*Table S1.* Package names versions and references used in the data analysis.

| Package name | Version | Reference |
| --- | --- | --- |
| lme4 | 1.1-23 | Bates, Machler, Bolker, and Walker (2015) |
| metafor | 2.4-0 | Viechtbauer (2010) |
| lavaan | 0.6-6 | Rosseel (2012) |
| lavaan.survey | 1.1.3.1 | Oberski (2014) |
| semTools | 0.5-3 | Jorgensen et al. (2018) |

## Exploratory analyses with bust trials

### Information seeking

Overall, across the five experiments, subjects sought information on nearly half of the bust trials (*M* = 43%, *SE* = 9%). The meta-analysis of the mean proportion of trials on which information was sought revealed significant heterogeneity between the experiments, *I^2^* = 89%. Observing the proportion of information seeking across the five experiments reveals that information seeking was highest (*M* = 60%, *SE* = 7%) in Experiment 1 when it incurred no cost and lowest (*M* = 14%, *SE* = 4%) in Experiment 2 when it incurred a monetary cost, and moderate in Experiments 3 (*M* = 55%, *SE* = 6%), 4 (*M* = 45%, *SE* = 8%), and 5 (*M* = 44%, *SE* = 8%). The probability of information seeking was significantly different from zero across all five experiments (all *p*s < .001).

### Emotional experience

We did not expect to find that that information seeking on bust trials would generally lead participants to feel worse. This is because, in contrast to bank trials, we expected participants to feel bad only when they learned that they were close to the balloon’s limit – a near miss. Otherwise, participants would be learning that even if they had changed their response a little, the outcome would have remained the same, leading to a neutral emotional response or even relief. We nonetheless sought to replicate our analyses from the bank trials. We conducted mixed-effects modeling predicting participant’s emotion change ratings from information-seeking (-1 = information not sought; 1 = information sought), pump value, and missed opportunity, i.e. the difference between the number of pumps and the balloon’s limit (Table S2, Step 1). Participants felt significantly worse after receiving information about the balloon’s limit than after rejecting that information, *b* = -0.082, *z* = -2.01, *p* = .045, 95% CI [-0.161, -0.002], but to a lesser extent than in bank trials and with some heterogeneity between studies (*I^2^* = 57%, see Figure S1a for average emotion change ratings on bust trials for each participant in each experiment after seeking and not seeking information). There was no significant main effect of the pump’s value, but there was a positive main effect of the missed opportunity, suggesting that overall, participants felt significantly worse when the missed opportunity was large.

To determine whether the missed opportunity has similar effects after bust as bank trials, we added the interaction terms with the information seeking to the model (Step 2). The negative main effect of information seeking did not remain significant after controlling for the interactions between information seeking and pump value and the missed opportunity. Importantly, however, the analysis showed that there was a negative interaction between information seeking and missed opportunity (Figure S1b), *b* = -0.063, *z* = -2.77, *p* = .006, 95% CI [-0.107, -0.018], *I^2^* = 76%. Simple slopes analyses suggest that when participants sought information, there was a significant effect of missed opportunity: the larger the missed opportunity, the worse they felt, *b* = -0.120, *z* = -2.29, *p* = .022, 95% CI [-0.223, -0.017], *I^2^* = 91%. Participants felt bad when there was a near miss, suggesting that they may have experienced regret when they learned that they could have banked if they had pumped a little less. In contrast, they felt better when the limit was further from the number of pumps they made, suggesting that they experienced relief because they would not have won, even if they had pumped a little less. When participants did not seek information, there was no significant association between missed opportunity and emotion change ratings *b* = -0.001, *z* = -0.08, *p* = .937, 95% CI [-0.015, 0.014], *I^2^* = 3%. The interaction between information seeking and pump value was not significant on bust trials. These findings were broadly in line with the results from bank trials reported in the manuscript. See Figure S1b for emotion change ratings by missed opportunity for the five experiments and the integrated result.

*Table S2.* Integrated results of models predicting emotion change ratings from information seeking, pump value, and the missed opportunity. Step 1 includes the main effects. Step 2 includes the main effects and interactions between information seeking and both pump value and missed opportunity.

|  | Step 1: Main effects | | | | |  | Step 2: Main effects and interactions | | | | |
| --- | --- | --- | --- | --- | --- | --- | --- | --- | --- | --- | --- |
|  | *b* | *(SE)* | *z* | *p* | *I^2^* |  | *b* | *(SE)* | *z* | *p* | *I^2^* |
| Information seeking (IS) | -0.082 | (0.041) | -2.01 | .045 | 57% | | -0.066 | (0.050) | -1.31 | 0.19 | 69% |
| Pump value (PV) | -0.017 | (0.041) | -0.42 | .677 | 49% |  | -0.006 | (0.044) | -0.13 | .899 | 53% |
| Missed opportunity (MO) | -0.041 | (0.012) | -3.53 | < .001 | 0% |  | -0.056 | (0.028) | -2.03 | .042 | 83% |
| IS*PV |  |  |  |  |  |  | 0.043 | (0.03) | 1.45 | .148 | 0% |
| IS*MO |  |  |  |  |  |  | -0.063 | (0.023) | -2.77 | .006 | 76% |

*Note.* SE = standard error.


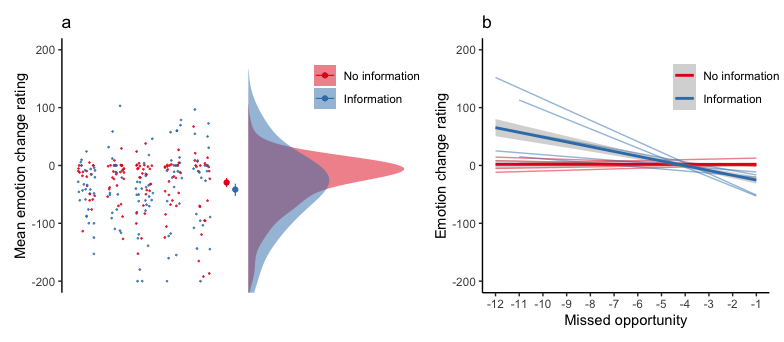


*Figure S1.* Effects of information on emotion change ratings on bust trials. a. In the raincloud plot, each column of small dots represents one experiment. Each small dot represents a participant’s mean emotion change rating on trials in which they did (blue) and did not (red) seek information. The larger dots represent the means for the whole sample, error bars represent 95% confidence intervals. b. Linear effects of missed opportunity on emotion change ratings for information and no information. Thicker lines represent the overall effects, thinner lines represent each of the five experiments. The shaded area around the thicker line represents the standard error. Emotion change ratings were mean centered within participants before plotting.

### Downstream effects of information seeking

To determine whether seeking information on bust trials affected participants’ downstream performance, we conducted a multilevel path analysis (structural equation model). We modelled a path from information seeking via emotion change and behavioral adjustment on the next trial (positive value means becoming more risky after the current bust trial) to the outcome of the next trial (both the probability of a bank outcome, and in terms of the number of points participants banked). Like the previous analyses, we controlled for the missed opportunity and the pump value as well as the interaction between these and information seeking (these control variables are not shown in the path diagram, see Table S6 for a table of all the model parameters).


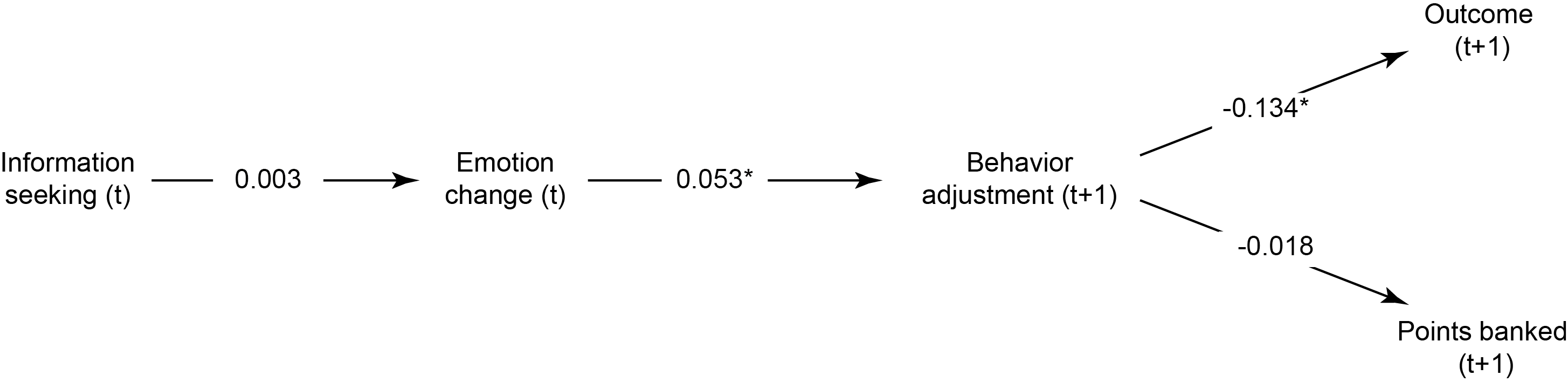


*Figure* *S2*. Path analysis demonstrating the effect of information seeking on emotion change ratings, behavioral adjustment and next trial performance. The main effects of missed opportunity and pump value were included as control variables along with their interactions with information seeking but are not shown on the path diagram.

Consistent with the previous analysis, information seeking did not have a significant negative effect on emotion change ratings overall, *b* = 0.003, *z* = 0.070, *p* = .944, 95% CI [-0.087, 0.094], *I^2^* = 8%, but it was qualified by a negative interaction with the missed opportunity, *b* = -0.247, *z* = -2.40, *p* = .017, 95% CI [-0.540, -0.045], *I^2^* = 49%. People felt worse after near misses. The next step of the path was between the emotion change rating and the adjustment of the number of pumps made on the next trial. The worse participants felt, the more conservative they became on the next trial, *b* = 0.053, *z* = 2.25, *p* = .024, 95% CI [0.007, 0.100], *I^2^* = 0%. Positive behavioral adjustment (becoming more risky) had a negative effect on the probability of a bank outcome, *b* = -0.134, *z* = -17.47, *p* < .001, 95% CI [-0.150, -0.119], *I^2^* = 0%, but no significant effect on the number of points banked, *b* = -0.018, *z* = -1.20, *p* = .229, 95% CI [-0.047, 0.011], *I^2^* = 0%, on the next trial. We also tested the significance of the mediation effect from information seeking to the next trial outcome and the next trial points and found that neither of the mediation effects had was significant, suggesting that information seeking did not have significant downstream effects on performance (see Table S5).

## Predicting information seeking

To determine which factors led participants to seek information, we examined the effects of a number of trial-level variables on the probability of seeking information about the balloon’s limit using a logistic mixed effects model with maximal random effects structure. Information seeking was predicted from the outcome (bust = -1, bank = 1), the number of pumps, and the pump value. Parameter estimates (*b*) are expressed as log odds. There was a significant main effect of the trial outcome, *b* = 0.196, *z* = 2.51, *p* = .012, 95% CI [0.043, 0.035], *I^2^* = 39%, participants were more likely to seek information after a bank outcome than after a bust outcome. There was a main effect of the number of pumps, *b* = 0.080, *z* = 3.56, *p* < .001, 95% CI [0.036, 0.125], *I^2^* = 0%, the more pumps participants made, the more likely they were to seek information. The main effect of pump value and interactions between the outcome and both the number of pumps and the pump value were not significant (see Table S3).

*Table S3*. Model parameters for the model predicting information seeking.

|  | *b* | *(SE)* | *z* | *p* | *I^2^* |
| --- | --- | --- | --- | --- | --- |
| Outcome (O) | 0.196 | (0.078) | 2.51 | .012 | 39% |
| Number of pumps (NP) | 0.080 | (0.023) | 3.56 | < .001 | 0% |
| Pump value (PV) | 0.460 | (0.308) | 1.49 | .135 | 90% |
| O*NP | -0.036 | (0.022) | -1.64 | .100 | 0% |
| O*PV | 0.023 | (0.093) | 0.24 | .807 | 22% |

*Note.* SE = standard error

We have suggested that participants sought information on bank trials despite expecting negative emotional experiences. However, it is plausible that participants were motivated by the possibility of learning that they were ‘just right’, a positive experience that happens occasionally after seeking information. If this was the case, we would predict a positive relationship between past emotion change ratings and information seeking – participants would seek more after feeling good, and less after feeling bad. To test this, we conducted an exploratory analysis in which we predicted information seeking on trial *t+1* from emotion change ratings on trial *t* using a generalized linear mixed effects model. The model only included bank trials in which participants sought information on trial *t*, and then banked again on *t+1*. We found no relationship between emotion change ratings and later information seeking, *b* = -0.000, *z* = -0.17, *p* = .866, 95% CI [-0.003, 0.003], *I^2^* = 14%, suggesting that participants were neither put off by negative experiences, nor encouraged by positive experiences.

**Optimal parameter values in mathematical modelling**

Figure S3 shows the relationship between parameter *α_i_* (information seekers) and *α_o_* (outcome observers) and point gain in comparison to the baseline model (Gaussian random walk model). As can be seen, both information seekers and outcome observers can optimize their performance when the adjustment parameter is less than 0.5. Outcome observers exhibited a large performance decrease when *α_o_* is very high; this is because, in that case, these hypothetical participants typically pump near the maximum number (i.e. 12) after they win and minimum number (i.e. 1) after they lose. In reality, participants who use such an extreme strategy are rarely seen.


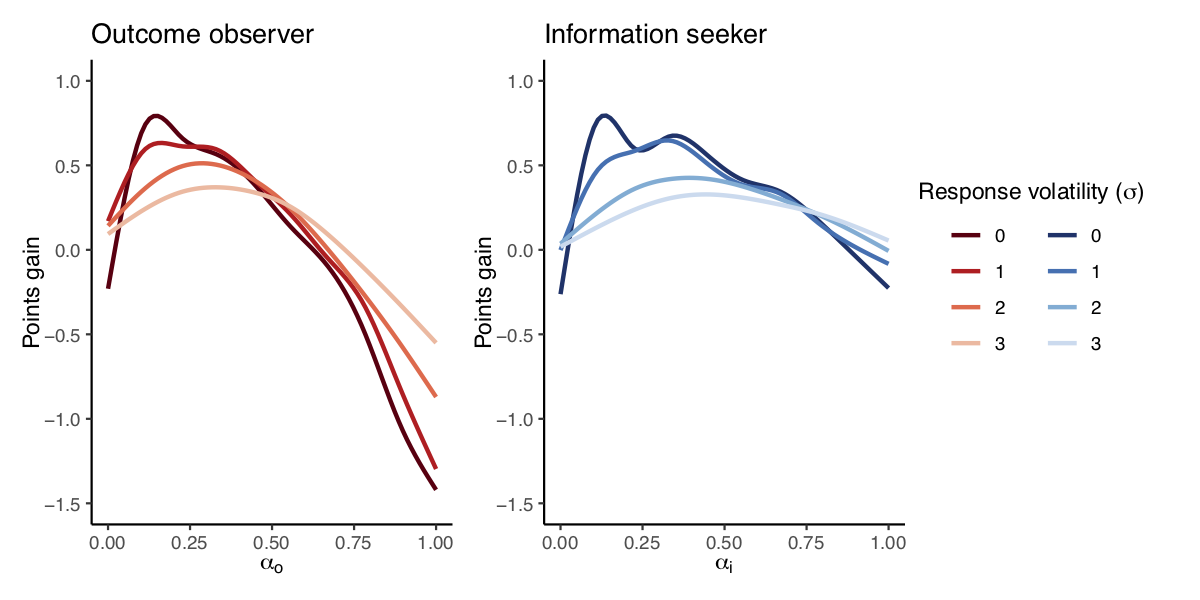


*Figure S3.* Gain from the baseline model by parameters *α_o_* and *α_i_* and response volatility (*σ*).

Table S4 shows the optimal parameter values to optimize the performance for each type of hypothetical participant. These values were computed by fitting generalized additive models to the simulation data for each type of hypothetical participant and each level of response volatility. These models were then used to predict the points gain at 1000 regular intervals along the *α_o_* and *α_i_* parameters. For each interval, the difference between the baseline model and both the outcome observer and information seeker models was calculated (see Figure S3). The *α_o_* and *α_i_* parameters at the maximum predicted gain was taken to be the optimal parameter value for each level of response volatility.

*Table S4.* Optimal parameter values for each type of hypothetical participant and each level of response volatility.

| Response volatility (*σ*) | *α_o_* | *α_i_* |
| --- | --- | --- |
| 0 | 0.145 | 0.132 |
| 1 | 0.160 | 0.324 |
| 2 | 0.286 | 0.393 |
| 3 | 0.326 | 0.442 |

## Replication study

The results of the replication study with only one critical trial closely replicate the results of the main study.

*
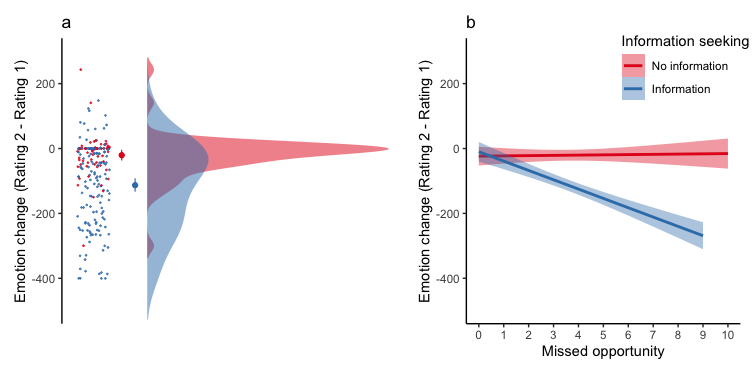
*

*Figure S4.* Effects of information on emotion change in the replication study. a. In the raincloud plot, participants’ emotions became more negative after seeking information than not doing so. Each dot represents a participant’s mean emotion change rating on trials in which they did (blue) and did not (red) seek information. The larger blue dots represent the group means, error bars represent 95% confidence intervals. b. Linear effects of missed opportunity on emotion change for information and no information, shaded areas represent standard error.

The main analysis and the above figure present the results with the difference between the first and second ratings as the dependent variable. The analyses were repeated with the second rating alone as the dependent vaiable. The results remain unchanged. Participants who sought information reported significantly lower emotion ratings than those who did not do so (*M* = 38.82; *SE* = 10.40) than those who did not seek information (*M* = 115.37; *SE* = 8.67), *t*(199.95) = -5.65, *p* < .001, *d* = -0.67, 95% CI [-0.97, -0.37]. Linear regression models predicting both the emotion rating change and the second emotion rating showed significant effects of information seeking when controlling for the number of pumps made, emotion change: *b* = -0.397, *t* = -5.83, *p* < .001, 95% CI [-0.532, -0.263]; second rating: *b* = -0.342, *t* = -5.00, *p* < .001, 95% CI [-0.477, -0.207].

## Parameter estimate plots

Model parameter estimates for each experiment and the meta-analysis. *I^2^* represents the heterogeneity of the effects across experiments; *I^2^* = 50% or more indicate substantial heterogeneity (Borenstein, Hedges, Higgins, & Rothstein, 2009).

*
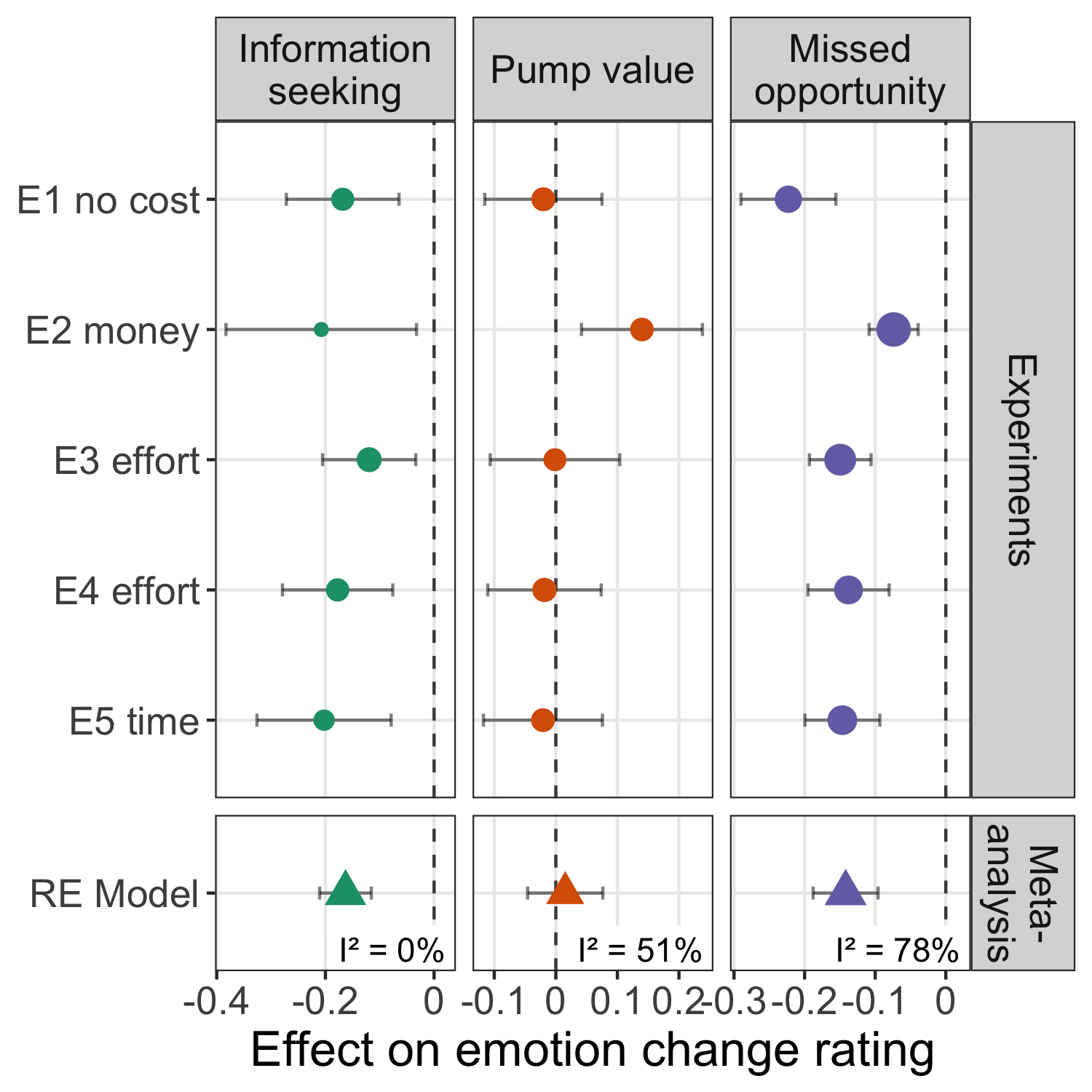
*

*Figure S5*. Emotional experience on bank trials (Step 1).


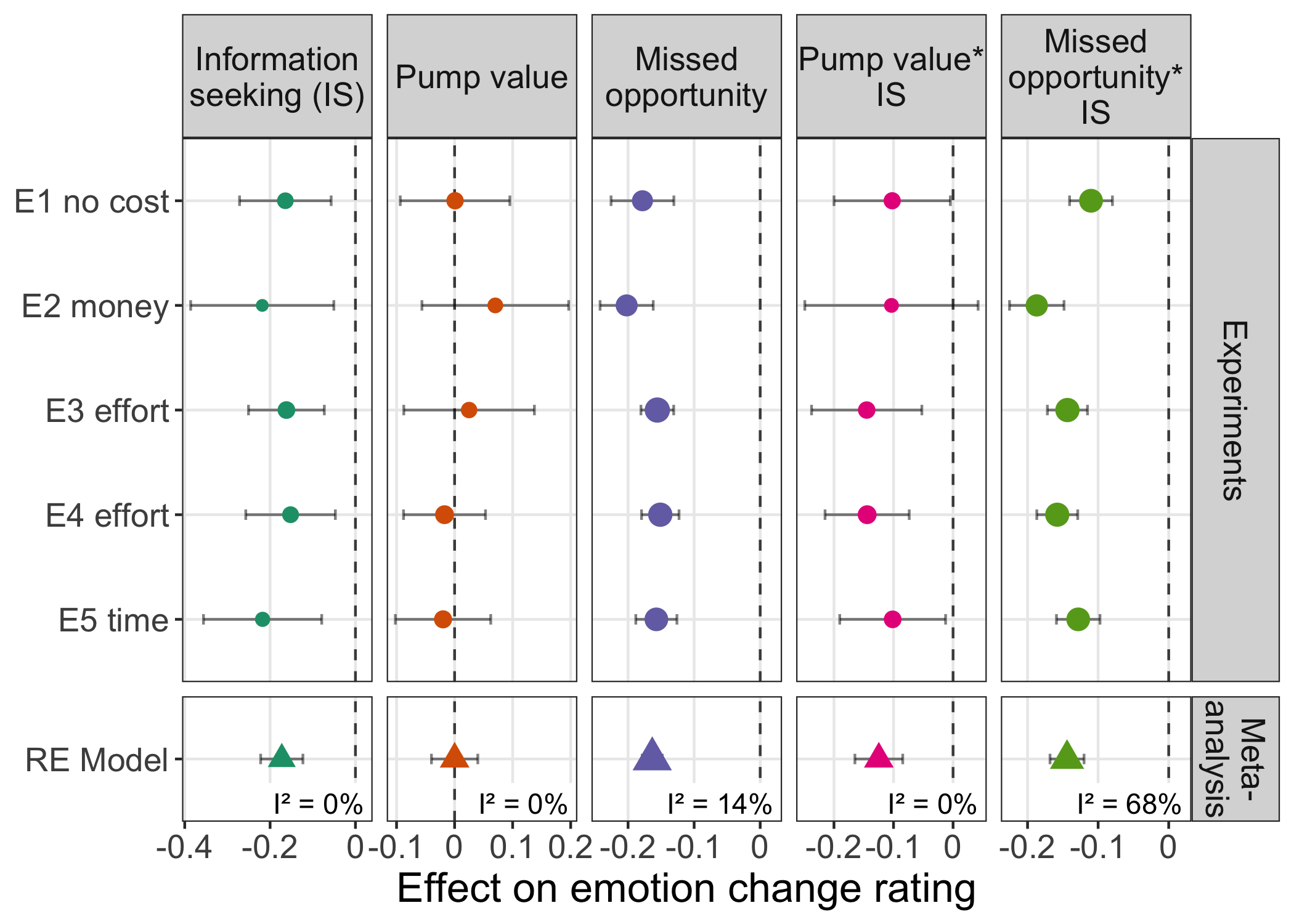


*Figure S6*. Emotional experience on bank trials (Step 2).


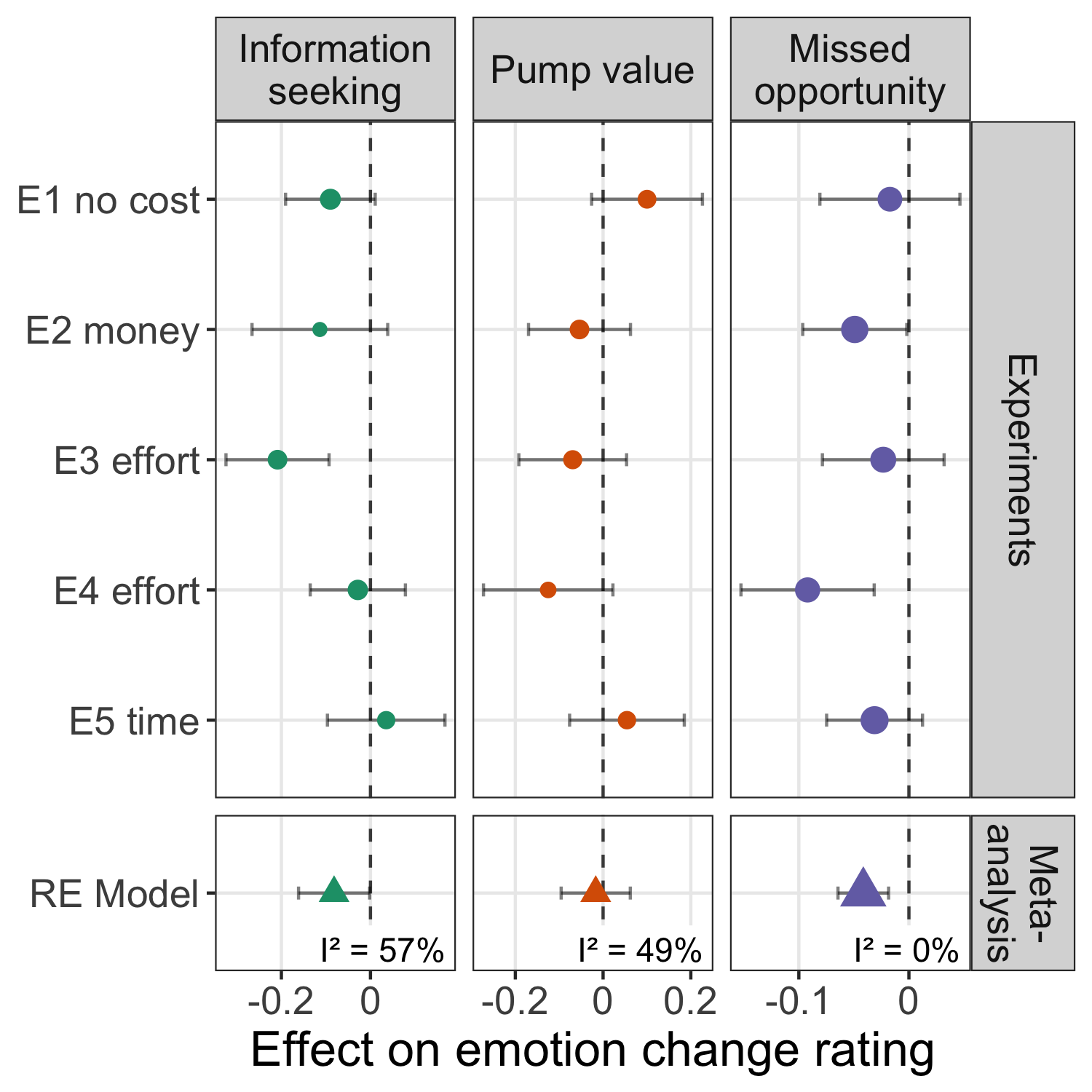


*Figure S7*. Emotional experience on bust trials (Step 1).


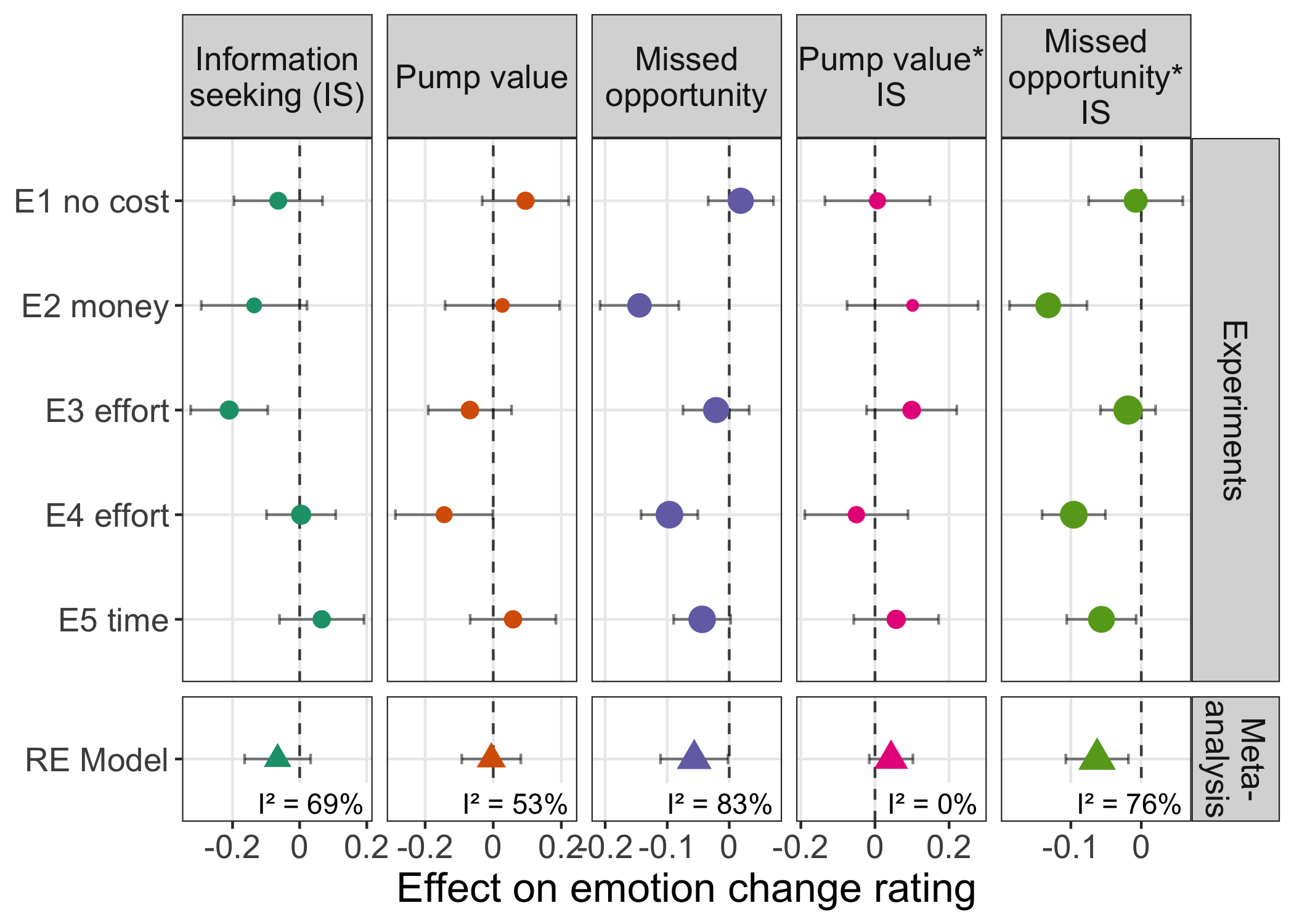


*Figure S8*. Emotional experience on bust trials (Step 2).

**Path analysis parameter tables**

All parameter estimates from the multi-level path models are tabulted on the following pages. The statistics reported in the paper are from the unsaturated models. Saturated model parameters are also included.

*Table S5.* Parameter estimates from the unsaturated path model for bank trials.

|  | *b* | *SE* | *95% CI* | *z* | *p* | *I^2^* |
| --- | --- | --- | --- | --- | --- | --- |
| **Model paths** |  |  |  |  |  |  |
| Information seeking (t)→Emotion change (t) | -0.191 | 0.031 | [-0.252, -0.130] | -6.13 | < .001 | 0% |
| Emotion change (t)→Behavior adjustment (t+1) | -0.089 | 0.018 | [-0.125, -0.053] | -4.86 | < .001 | 0% |
| Behavior adjustment (t+1)→Outcome (t+1) | -0.157 | 0.007 | [-0.170, -0.143] | -22.68 | < .001 | 0% |
| Behavior adjustment (t+1)→Points (t+1) | -0.040 | 0.014 | [-0.069, -0.012] | -2.81 | .005 | 0% |
| **Control paths** |  |  |  |  |  |  |
| IS*Missed opportunity (t)→Emotion change (t) | -0.656 | 0.062 | [-0.777, -0.536] | -10.65 | < .001 | 0% |
| IS*Missed opportunity (t)→Behavior adjustment (t+1) | 0.020 | 0.034 | [-0.046, 0.087] | 0.60 | .549 | 0% |
| IS*Missed opportunity (t)→Outcome (t+1) | 0.025 | 0.013 | [ 0.000, 0.051] | 2.00 | .046 | 22% |
| IS*Missed opportunity (t)→Points (t+1) | 0.047 | 0.021 | [ 0.005, 0.088] | 2.20 | .028 | 30% |
| IS*Pump value (t)→Emotion change (t) | -0.137 | 0.026 | [-0.188, -0.087] | -5.33 | < .001 | 0% |
| IS*Pump value (t)→Behavior adjustment (t+1) | 0.029 | 0.045 | [-0.060, 0.117] | 0.63 | .527 | 0% |
| IS*Pump value (t)→Outcome (t+1) | -0.021 | 0.017 | [-0.053, 0.011] | -1.27 | .204 | 31% |
| IS*Pump value (t)→Points (t+1) | -0.025 | 0.022 | [-0.068, 0.018] | -1.12 | .262 | 0% |
| Missed opportunity (t)→Emotion change (t) | -0.715 | 0.058 | [-0.830, -0.601] | -12.26 | < .001 | 0% |
| Missed opportunity (t)→Behavior adjustment (t+1) | 0.263 | 0.023 | [ 0.218, 0.308] | 11.39 | < .001 | 0% |
| Missed opportunity (t)→Outcome (t+1) | 0.046 | 0.018 | [ 0.011, 0.080] | 2.60 | .009 | 57% |
| Missed opportunity (t)→Points (t+1) | -0.022 | 0.018 | [-0.058, 0.013] | -1.24 | .214 | 0% |
| Pump value (t)→Emotion change (t) | -0.018 | 0.026 | [-0.070, 0.033] | -0.69 | .49 | 0% |
| Pump value (t)→Behavior adjustment (t+1) | 0.195 | 0.047 | [ 0.102, 0.287] | 4.13 | < .001 | 0% |
| Pump value (t)→Outcome (t+1) | 0.027 | 0.017 | [-0.007, 0.061] | 1.54 | .123 | 38% |
| Pump value (t)→Points (t+1) | 0.015 | 0.022 | [-0.028, 0.058] | 0.67 | .506 | 0% |
| **Covariance** |  |  |  |  |  |  |
| Points (t+1)←→Outcome (t+1) | 0.230 | 0.008 | [ 0.214, 0.245] | 28.21 | < .001 | 0% |
| **Mediated paths** |  |  |  |  |  |  |
| IS → EC → BA → Outcome | -0.003 | 0.001 | [-0.004, -0.001] | -3.41 | < .001 | 0% |
| IS → EC → BA → Points | 0.000 | 0.000 | [-0.001, 0.000] | -1.43 | .152 | 0% |

*Note.* SE = standard error; 95% CI = 95% confidence intervals.

*Table S6.* Parameter estimates from the saturated path model for bank trials.

|  | *b* | *SE* | *95% CI* | *z* | *p* | *I^2^* |
| --- | --- | --- | --- | --- | --- | --- |
| **Model paths** |  |  |  |  |  |  |
| Information seeking (t)→Emotion change (t) | -0.191 | 0.031 | [-0.252, -0.130] | -6.13 | < .001 | 0% |
| Emotion change (t)→Behavior adjustment (t+1) | -0.090 | 0.019 | [-0.127, -0.053] | -4.81 | < .001 | 0% |
| Behavior adjustment (t+1)→Outcome (t+1) | -0.157 | 0.007 | [-0.171, -0.144] | -22.68 | < .001 | 0% |
| Behavior adjustment (t+1)→Points (t+1) | -0.041 | 0.015 | [-0.069, -0.012] | -2.76 | .006 | 0% |
| **Control paths** |  |  |  |  |  |  |
| Information seeking (t)→Behavior adjustment (t+1) | 0.000 | 0.015 | [-0.028, 0.029] | 0.01 | .995 | 0% |
| Information seeking (t)→Outcome (t+1) | -0.003 | 0.012 | [-0.026, 0.020] | -0.28 | .781 | 33% |
| Information seeking (t)→Points (t+1) | 0.016 | 0.022 | [-0.026, 0.058] | 0.74 | .457 | 60% |
| Emotion change (t)→Outcome (t+1) | -0.010 | 0.011 | [-0.031, 0.011] | -0.96 | .337 | 0% |
| Emotion change (t)→Points (t+1) | 0.005 | 0.014 | [-0.023, 0.034] | 0.38 | .703 | 0% |
| IS*Missed opportunity (t)→Emotion change (t) | -0.656 | 0.062 | [-0.777, -0.536] | -10.65 | < .001 | 0% |
| IS*Missed opportunity (t)→Behavior adjustment (t+1) | 0.021 | 0.035 | [-0.047, 0.089] | 0.61 | .541 | 0% |
| IS*Missed opportunity (t)→Outcome (t+1) | 0.020 | 0.013 | [-0.005, 0.046] | 1.57 | .117 | 0% |
| IS*Missed opportunity (t)→Points (t+1) | 0.057 | 0.020 | [ 0.018, 0.097] | 2.85 | .004 | 4% |
| IS*Pump value (t)→Emotion change (t) | -0.137 | 0.026 | [-0.188, -0.087] | -5.33 | < .001 | 0% |
| IS*Pump value (t)→Behavior adjustment (t+1) | 0.030 | 0.046 | [-0.059, 0.120] | 0.66 | .507 | 0% |
| IS*Pump value (t)→Outcome (t+1) | -0.020 | 0.016 | [-0.052, 0.012] | -1.24 | .216 | 25% |
| IS*Pump value (t)→Points (t+1) | -0.025 | 0.023 | [-0.069, 0.019] | -1.10 | .27 | 0% |
| Missed opportunity (t)→Emotion change (t) | -0.715 | 0.058 | [-0.830, -0.601] | -12.26 | < .001 | 0% |
| Missed opportunity (t)→Behavior adjustment (t+1) | 0.262 | 0.024 | [ 0.215, 0.309] | 10.89 | < .001 | 6% |
| Missed opportunity (t)→Outcome (t+1) | 0.041 | 0.019 | [ 0.003, 0.078] | 2.14 | .032 | 44% |
| Missed opportunity (t)→Points (t+1) | -0.018 | 0.020 | [-0.058, 0.022] | -0.89 | .373 | 0% |
| Pump value (t)→Emotion change (t) | -0.018 | 0.026 | [-0.070, 0.033] | -0.69 | .49 | 0% |
| Pump value (t)→Behavior adjustment (t+1) | 0.193 | 0.047 | [ 0.101, 0.286] | 4.11 | < .001 | 0% |
| Pump value (t)→Outcome (t+1) | 0.027 | 0.017 | [-0.007, 0.061] | 1.58 | .114 | 34% |
| Pump value (t)→Points (t+1) | 0.012 | 0.022 | [-0.032, 0.056] | 0.55 | .584 | 0% |
| **Covariance** |  |  |  |  |  |  |
| Points (t+1)←→Outcome (t+1) | 0.230 | 0.008 | [ 0.214, 0.246] | 28.46 | < .001 | 0% |
| **Mediated paths** |  |  |  |  |  |  |
| IS → EC → BA → Outcome | -0.003 | 0.001 | [-0.004, -0.001] | -3.02 | .003 | 0% |
| IS → EC → BA → Points | 0.000 | 0.000 | [-0.001, 0.000] | -1.31 | .191 | 0% |

*Note.* SE = standard error; 95% CI = 95% confidence intervals.

*Table S7.* Parameter estimates from the unsaturated path model for bust trials.

|  | *b* | *SE* | *95% CI* | *z* | *p* | *I^2^* |
| --- | --- | --- | --- | --- | --- | --- |
| **Model paths** |  |  |  |  |  |  |
| Information seeking (t)→Emotion change (t) | 0.003 | 0.046 | [-0.087, 0.094] | 0.07 | .944 | 8% |
| Emotion change (t)→Behavior adjustment (t+1) | 0.053 | 0.024 | [ 0.007, 0.100] | 2.25 | .024 | 0% |
| Behavior adjustment (t+1)→Outcome (t+1) | -0.134 | 0.008 | [-0.150, -0.119] | -17.47 | < .001 | 0% |
| Behavior adjustment (t+1)→Points (t+1) | -0.018 | 0.015 | [-0.047, 0.011] | -1.20 | .229 | 0% |
| **Control paths** |  |  |  |  |  |  |
| IS*Missed opportunity (t)→Emotion change (t) | -0.247 | 0.103 | [-0.450, -0.045] | -2.40 | .017 | 49% |
| IS*Missed opportunity (t)→Behavior adjustment (t+1) | 0.088 | 0.070 | [-0.050, 0.226] | 1.26 | .209 | 0% |
| IS*Missed opportunity (t)→Outcome (t+1) | 0.006 | 0.024 | [-0.041, 0.053] | 0.26 | .793 | 21% |
| IS*Missed opportunity (t)→Points (t+1) | 0.044 | 0.046 | [-0.047, 0.134] | 0.94 | .345 | 54% |
| IS*Pump value (t)→Emotion change (t) | 0.071 | 0.033 | [ 0.007, 0.135] | 2.16 | .031 | 0% |
| IS*Pump value (t)→Behavior adjustment (t+1) | 0.046 | 0.056 | [-0.064, 0.155] | 0.82 | .411 | 0% |
| IS*Pump value (t)→Outcome (t+1) | -0.009 | 0.025 | [-0.057, 0.039] | -0.38 | .706 | 29% |
| IS*Pump value (t)→Points (t+1) | -0.024 | 0.030 | [-0.083, 0.035] | -0.80 | .423 | 0% |
| Missed opportunity (t)→Emotion change (t) | -0.199 | 0.068 | [-0.331, -0.066] | -2.94 | .003 | 0% |
| Missed opportunity (t)→Behavior adjustment (t+1) | 0.781 | 0.056 | [ 0.672, 0.889] | 14.06 | < .001 | 0% |
| Missed opportunity (t)→Outcome (t+1) | 0.140 | 0.021 | [ 0.099, 0.180] | 6.78 | < .001 | 0% |
| Missed opportunity (t)→Points (t+1) | 0.064 | 0.032 | [ 0.001, 0.126] | 2.01 | .045 | 0% |
| Pump value (t)→Emotion change (t) | 0.058 | 0.042 | [-0.024, 0.141] | 1.38 | .166 | 30% |
| Pump value (t)→Behavior adjustment (t+1) | 0.338 | 0.075 | [ 0.191, 0.485] | 4.52 | < .001 | 36% |
| Pump value (t)→Outcome (t+1) | 0.035 | 0.019 | [-0.002, 0.072] | 1.84 | .065 | 0% |
| Pump value (t)→Points (t+1) | 0.051 | 0.029 | [-0.006, 0.108] | 1.76 | .078 | 0% |
| **Covariance** |  |  |  |  |  |  |
| Points (t+1)←→Outcome (t+1) | 0.248 | 0.010 | [ 0.229, 0.267] | 25.26 | < .001 | 19% |
| **Mediated paths** |  |  |  |  |  |  |
| IS → EC → BA → Outcome | 0.000 | 0.000 | [ 0.000, 0.001] | 0.26 | .798 | 0% |
| IS → EC → BA → Points | 0.000 | 0.000 | [ 0.000, 0.000] | 0.26 | .796 | 0% |

*Note.* SE = standard error; 95% CI = 95% confidence intervals.

*Table S8.* Parameter estimates from the saturated path model for bust trials.

|  | *b* | *SE* | *95% CI* | *z* | *p* | *I2* |
| --- | --- | --- | --- | --- | --- | --- |
| **Model paths** |  |  |  |  |  |  |
| Information seeking (t)→Emotion change (t) | 0.003 | 0.046 | [-0.087, 0.094] | 0.07 | .944 | 8% |
| Emotion change (t)→Behavior adjustment (t+1) | 0.049 | 0.025 | [ 0.001, 0.098] | 2.01 | .045 | 0% |
| Behavior adjustment (t+1)→Outcome (t+1) | -0.136 | 0.008 | [-0.151, -0.121] | -17.77 | < .001 | 0% |
| Behavior adjustment (t+1)→Points (t+1) | -0.020 | 0.015 | [-0.049, 0.009] | -1.33 | .184 | 0% |
| **Control paths** |  |  |  |  |  |  |
| Information seeking (t)→Behavior adjustment (t+1) | -0.068 | 0.021 | [-0.109, -0.027] | -3.25 | .001 | 0% |
| Information seeking (t)→Outcome (t+1) | -0.019 | 0.011 | [-0.041, 0.002] | -1.75 | .08 | 28% |
| Information seeking (t)→Points (t+1) | -0.033 | 0.021 | [-0.073, 0.008] | -1.58 | .114 | 47% |
| Emotion change (t)→Outcome (t+1) | 0.003 | 0.012 | [-0.021, 0.028] | 0.26 | .797 | 36% |
| Emotion change (t)→Points (t+1) | 0.008 | 0.017 | [-0.025, 0.040] | 0.45 | .655 | 0% |
| IS*Missed opportunity (t)→Emotion change (t) | -0.247 | 0.103 | [-0.450, -0.045] | -2.40 | .017 | 49% |
| IS*Missed opportunity (t)→Behavior adjustment (t+1) | 0.074 | 0.070 | [-0.063, 0.211] | 1.06 | .289 | 0% |
| IS*Missed opportunity (t)→Outcome (t+1) | 0.005 | 0.025 | [-0.043, 0.053] | 0.20 | .843 | 15% |
| IS*Missed opportunity (t)→Points (t+1) | 0.040 | 0.045 | [-0.048, 0.128] | 0.89 | .373 | 48% |
| IS*Pump value (t)→Emotion change (t) | 0.071 | 0.033 | [ 0.007, 0.135] | 2.16 | .031 | 0% |
| IS*Pump value (t)→Behavior adjustment (t+1) | 0.066 | 0.058 | [-0.046, 0.179] | 1.15 | .249 | 0% |
| IS*Pump value (t)→Outcome (t+1) | -0.007 | 0.025 | [-0.056, 0.042] | -0.29 | .775 | 28% |
| IS*Pump value (t)→Points (t+1) | -0.029 | 0.030 | [-0.089, 0.030] | -0.96 | .336 | 0% |
| Missed opportunity (t)→Emotion change (t) | -0.199 | 0.068 | [-0.331, -0.066] | -2.94 | .003 | 0% |
| Missed opportunity (t)→Behavior adjustment (t+1) | 0.763 | 0.060 | [ 0.644, 0.881] | 12.63 | < .001 | 14% |
| Missed opportunity (t)→Outcome (t+1) | 0.142 | 0.022 | [ 0.099, 0.184] | 6.57 | < .001 | 0% |
| Missed opportunity (t)→Points (t+1) | 0.064 | 0.033 | [-0.002, 0.129] | 1.91 | .056 | 0% |
| Pump value (t)→Emotion change (t) | 0.058 | 0.042 | [-0.024, 0.141] | 1.38 | .166 | 30% |
| Pump value (t)→Behavior adjustment (t+1) | 0.359 | 0.084 | [ 0.194, 0.524] | 4.27 | < .001 | 48% |
| Pump value (t)→Outcome (t+1) | 0.037 | 0.019 | [-0.001, 0.075] | 1.92 | .055 | 0% |
| Pump value (t)→Points (t+1) | 0.050 | 0.030 | [-0.009, 0.108] | 1.67 | .095 | 0% |
| **Covariance** |  |  |  |  |  |  |
| Points (t+1)←→Outcome (t+1) | 0.247 | 0.010 | [ 0.228, 0.267] | 24.87 | < .001 | 20% |
| **Mediated paths** |  |  |  |  |  |  |
| IS → EC → BA → Outcome | 0.000 | 0.000 | [ 0.000, 0.001] | 0.27 | .788 | 0% |
| IS → EC → BA → Points | 0.000 | 0.000 | [ 0.000, 0.000] | 0.28 | .781 | 0% |

*Note.* SE = standard error; 95% CI = 95% confidence intervals.

# References

Bates, D., Machler, M., Bolker, B. M., & Walker, S. C. (2015). Fitting Linear Mixed-Effects Models Using lme4. *Journal of Statistical Software, 67*(1), 1-48. Retrieved from <Go to ISI>://WOS:000365981400001.

Borenstein, M., Hedges, L. V., Higgins, J. P. T., & Rothstein, H. R. (2009). *Introduction to meta-analysis.* Chichester, United Kingdom: Wiley.

Jorgensen, T. D., Pornprasertmanit, S., Schoemann, A., Rosseel, Y., Miller, P., Quick, C., & Garnier-Villarreal, M. (2018). semTools: Useful tools for structural equation modeling. *R Package Version 0.5*.

Oberski, D. (2014). lavaan.survey: An R Package for Complex Survey Analysis of Structural Equation Models. *2014, 57*(1), 27. Retrieved from <https://www.jstatsoft.org/v057/i01>. doi:10.18637/jss.v057.i01

Rosseel, Y. (2012). Lavaan: An R package for structural equation modeling and more. *Journal of Statistical Software, 48*(2), 1-36.

Viechtbauer, W. (2010). Conducting meta-analyses in R with the metafor package. *Journal of Statistical Software, 36*, 1-48.
